# Increasing mobility while in hospital after hip fracture: even the best evidence may not translate to all national health services

**DOI:** 10.1186/s10195-026-00969-9

**Published:** 2026-10-15

**Authors:** Nicola Maffulli, Fabrizio Rivera, Giovanna Truda, Filippo Migliorini, Francesco Oliva

**Affiliations:** 1 Department of Trauma and Orthopaedic Surgery, Faculty of Medicine and Psychology, Sapienza University, Rome, Italy; 2 Centre for Sports and Exercise Medicine, Queen Mary University of London, London, UK; 3 Department of Orthopaedics, Ospedale Cottolengo, Turin, Italy; 4 Department of Sociology, University of Salerno, Fisciano, Italy; 5 Department of Trauma and Reconstructive Surgery, University Hospital Halle, Martin-Luther University Halle-Wittenberg, Ernst-Grube-Street 40, 06097 Halle (Saale), Germany; 6 Department of Life Sciences, Health, and Health Professions, Link Campus University, 00165 Rome, Italy; 7 Department of Orthopaedic and Trauma Surgery, Eifelklinik St.Brigida, 52152 Simmerath, Germany; 8 Department of Human Sciences and Promotion of the Quality of Life, San Raffaele Roma Open University, Rome, Italy

**Keywords:** Hip fracture, Rehabilitation, Early mobilisation, Physiotherapy, Discharge destination, Italy, National Health SystemEditorial

## Abstract

Hip fracture rehabilitation is strongly influenced by the healthcare system in which postoperative care is delivered. The WHiTE 15: INITIATE trial represents an important pragmatic attempt to determine whether increasing the frequency and duration of in-hospital physiotherapy after hip fracture improves days at home within 30 days of admission. However, the transferability of such evidence to the Italian National Health System is limited by structural, regulatory and sociocultural factors. Italy has one of the oldest populations in Europe, a high annual burden of proximal femoral fractures, a limited public physiotherapy workforce capacity and a care model in which transfer to inpatient rehabilitation facilities is frequently shaped by patient and family preferences rather than clinical indication alone. Current evidence supports early mobilisation and structured rehabilitation, but it does not establish that a single discharge pathway is universally superior across all healthcare systems. In Italy, direct discharge home is often constrained by insufficient community-based rehabilitation services and strong expectations for post-acute institutional care. Therefore, intensifying in-hospital physiotherapy alone may not increase the number of days at home unless discharge preferences, family support and community rehabilitation capacity are addressed simultaneously. Future research should evaluate Italian-specific discharge pathways, psychosocial determinants of rehabilitation preferences, cost-effectiveness of supported discharge models and policy strategies that align rehabilitation intensity with clinical need.

## Editorial

The recent WHiTE 15: INITIATE trial is a major effort: a two-arm, multicentre, cluster randomised controlled trial designed to determine whether increasing the frequency and duration of in-hospital physiotherapy after hip fracture improves the primary outcome of days at home within 30 days of admission (DAH30) [1] NICE first raised this issue in 2011 [2], and the 2018 James Lind Alliance made it a top priority [3]. Years later, a clear answer is still not available despite repeated pleas from patients, physicians, surgeons and health care professionals and policymakers [1]

Hip fractures in Italy are not just a clinical problem: they have become a social crisis in plain sight. With 24.7% of its population aged 65 years or older, the highest proportion in the European Union, Italy is the most aged nation on the continent [4]. The proportion of individuals aged at least 80 has reached 7.7%, more than double the figure recorded in 1991[4]. Approximately 90,000 proximal femoral fractures occur in Italy each year, a burden that continues to grow: the population is ageing rapidly, even though age-adjusted rates have dropped slightly [5]. More recent national data from the Italian National Healthcare Outcomes Programme (PNE-AGENAS) confirm the continuing high burden of hip fracture, with 96,777 hospital admissions for femoral neck fracture recorded in 2024 [5, 6]. Regional Italian data from Piedmont confirm this pattern: in a multicentric study of 5386 hip fracture patients treated across three hospitals in 2003, only 27.2% were discharged directly home after surgery, 48.1% were transferred to a rehabilitation facility and 24.7% were admitted to a nursing home [7]. Functional recovery was also modest: only 38.3% of patients returned to their prefracture walking ability without aids, while 35.2% required walking devices and 26.5% did not recover their ability to walk. These figures, now more than two decades old, should be interpreted as historical rather than representative of current national practice. As in the UK, one-year mortality following hip fracture ranges from 15 to 30%, and immobility is a primary driver of medical complications, institutionalisation and death [8–10].

Hip fracture is an event that can lead to serious complications in frail patients [11]. The current evidence strongly favours early mobilisation and discharge home as the optimal pathway after hip fracture surgery. A systematic review confirmed that early mobilisation is independently associated with improved functional outcomes and a reduced length of stay [12]. Randomised controlled trial evidence indicates that, in appropriately selected community-dwelling patients, early discharge followed by structured home rehabilitation achieves functional outcomes at least equivalent to those of conventional hospital-based rehabilitation, with greater improvements in mobility independence and balance confidence in some studies [13–16]. In a retrospective comparison including patients of varying medical complexity, short-term functional rehabilitation outcomes did not differ significantly between those who completed home-based and those who completed inpatient rehabilitation, even though the inpatient group had substantially higher comorbidity burden [17]. Such evidence arises from a relatively fit group of patients who lived at home before the fracture and had adequate social and clinical support. It does not reflect the full range of frail, often isolated patients seen in the Italian National Health System (Sistema Sanitario Nazionale – SSN). Crucially, the evidence on mortality does not support the assumption that home discharge is equivalent to structured post-acute rehabilitation. In a retrospective cohort study of 2208 hip fracture patients from the Bologna metropolitan area, patients who received no post-acute rehabilitation had a 6-month mortality risk more than twice that of patients receiving hospital-based rehabilitation (adjusted hazard ratio 2.19; 95% confidence interval 1.54–3.12; *p* < 0.001), and patients in private accredited inpatient rehabilitation facilities also had significantly higher mortality than those in hospital rehabilitation (hazard ratio 1.66; 95% confidence interval 1.54–1.79; *p* < 0.001) [19]. A large propensity-matched Canadian cohort study similarly evidenced that hip fracture patients discharged to the community had substantially higher rates of death or rehospitalisation within one year than those who received inpatient rehabilitation (27–42% versus 10–11%), despite lower overall healthcare costs [19]. The most recent Cochrane systematic review on multidisciplinary rehabilitation after hip fracture found moderate-certainty evidence that inpatient multidisciplinary rehabilitation reduces poor outcomes (death or deterioration in residential status), and concluded that the evidence base for supported discharge with home rehabilitation is of very low certainty, implying that no firm conclusions can be drawn regarding its equivalence to inpatient rehabilitation in either mortality or functional outcomes [20]. In the same Bologna cohort, 41% of patients were transferred to private inpatient rehabilitation facilities accredited by the SSN, 23.5% remained in hospital for inpatient rehabilitation, and only 35% received no post-acute institutional rehabilitation. These proportions illustrate that institutional transfer has become routine rather than a clinically selected exception; yet, far from justifying that practice as costless, the mortality data suggest that the absence of any structured rehabilitation carries a significant survival penalty.

Four clinically distinct post-operative pathways must be distinguished when interpreting this evidence. In Pathway A, the patient undergoes surgery, receives in-hospital rehabilitation within the geriatric or orthopaedic acute ward, and is subsequently discharged home. In Pathway B, the patient is discharged directly home from the acute ward, where rehabilitation is then delivered by a community-based multidisciplinary team. In Pathway C, the patient is discharged from the acute ward to an inpatient rehabilitation facility or nursing home for post-acute institutional rehabilitation. A fourth scenario, Pathway D, where patients leave the acute ward with no structured post-acute rehabilitation, is also clinically common, particularly in frail or socially isolated patients, and is associated with the worst outcomes [18]. The best available direct comparison of Pathways A and B comes from a Swedish RCT (n = 205, aged 70 or older, including patients with cognitive impairment) in which in-hospital geriatric multifactorial rehabilitation followed by home discharge (Pathway A) was compared with early supported discharge followed by geriatric interdisciplinary home rehabilitation for up to 10 weeks (Pathway B). This trial found no significant differences between the two arms at 3 or 12 months in walking ability, activities of daily living independence, falls, or hospital readmissions [21–23]. Pathway B was associated with a six-day shorter acute hospital stay. Mortality between discharge and 12 months did not differ significantly between the arms. However, this trial was conducted in Sweden, where robust community services, a high density of community-based physiotherapists and coordinated interdisciplinary home teams are the norm; these conditions do not currently exist in the Italian SSN, and the results cannot be transposed directly. The comparison between Pathways A and C and between different forms of Pathway C, is more complex. Within the broad category of Pathway C, there is an important distinction between inpatient rehabilitation facilities (IRF) with intensive multidisciplinary input and skilled nursing and residential facilities with lower rehabilitation intensity. A quasi-experimental study exploiting the closure of IRF units in US hospitals found that substituting IRF with skilled nursing facility care was associated with a 5.8% increase in 90-day mortality for hip fracture patients (95% confidence interval 2.5–9.0%), with the protective effect of IRF concentrated in the first 20 days post-discharge [24]. In the Italian context, the private accredited inpatient rehabilitation facilities (IRF) to which 41% of patients in the Bologna cohort were transferred represent Pathway C. However, the intensity of rehabilitation provided in those settings is not standardised and may be substantially lower than that achievable in a dedicated acute orthogeriatric ward [25, 26]. No randomised evidence directly compares Pathway A with Pathway C in the Italian SSN, and no prospective study has compared 30- or 90-day mortality across all four pathways. Therefore, current evidence does not establish that Pathway A, B, or D systematically provides better outcomes than a well-conducted Pathway C. The rationale for reorienting Italian practice is instead based on three considerations. First, Pathway D, without structured rehabilitation, is associated with substantially worse outcomes, including higher mortality [18, 19]. Second, within Pathway C, the quality and intensity of rehabilitation in private accredited facilities are variable and inadequately standardised; more intensive inpatient rehabilitation appears to be associated with better outcomes than lower-intensity nursing home care [24]. Third, the routine use of institutional rehabilitation as a default rather than a clinically indicated option consumes resources and may limit the development of community-based alternatives. Evidence from Sweden and smaller randomised studies from Australia and Sweden [13, 15–17] suggests that Pathway B can achieve outcomes comparable to Pathway A when supported by well-organised, adequately staffed and promptly delivered community rehabilitation services. However, such services are not currently scalable within the Italian SSN. The Italian SSN, unlike the NHS in England, allows patients and their families to opt for transfer to an inpatient rehabilitation facility (rehabilitative ward or Residenza Sanitaria Assistenziale, RSA) at public expense for up to 30 days, regardless of whether clinical criteria justify it. In practice, this means that patients’ preferences, and, crucially, the preferences of their families, override the medical recommendation for discharge home. This arrangement results from deep-rooted family and cultural attitudes. Italian society is characterised by strong family bonds and a marked tendency for families to regard transfer to a rehabilitation facility as providing greater safety and professional oversight than returning home, irrespective of clinical evidence [27]. The fear of falling, a common concern after hip fracture, makes families and patients more insistent on institutional care [28]. Patients who have experienced hip fractures frequently describe discharge as a moment of anxiety and vulnerability rather than relief, and many fear being a burden to their families [27]. Therefore, there is a real conflict between the scientific evidence about early discharge and what patients and families expect.

Psychosocial factors are powerful independent determinants of recovery after hip fracture [29]. Depression, low self-efficacy, limited social support and living alone each predict poorer functional outcomes at 12 months [30, 31] while higher patient activation, defined as patients’ knowledge, skills and confidence in managing their own health, is associated with substantially better functional independence [32]. In the Italian context, where family is frequently the primary caregiver, a patient’s sense of safety at home correlates strongly with perceived family capability. When families feel inadequately supported or when the home environment presents structural barriers, the demand for institutional care is unlikely to diminish, regardless of what clinical protocols recommend.

Can more intensive in-hospital physiotherapy translate into earlier discharge home? In Italy, there is a clear bottleneck. Italy has about 70,000 physiotherapists. Roughly 70% of them work privately and only about 21,000 work for the SSN [33]. This equates to approximately 35 SSN-employed physiotherapists per 100,000 population, a density substantially below the EU average of 136 physiotherapists per 100,000 inhabitants overall [34]. With approximately 90,000 hip fracture admissions per year, and assuming a mean acute hospital stay of 10 days [5], an approach analogous to the INITIATE intervention, offering at least one additional two-hour physiotherapy session per day in the first postoperative week, would require a minimum of one additional full-time physiotherapist per ward, continuously, across all treating hospitals. Assuming one additional two-hour physiotherapy session per day during the first postoperative week, this would correspond to approximately 14 additional physiotherapy hours per hip fracture admission, or approximately 1.26 million additional physiotherapy hours annually at the national level. This represents a substantial additional workforce requirement for the Italian SSN. A survey of Italian physiotherapists in 2023 found that recommendations for intensive rehabilitation and full weight-bearing after hip fracture did not meet the target adherence threshold of 70%, reflecting a gap between evidence-based guidance and actual practice, driven in part by insufficient staffing [26]. However, intensifying in-hospital physiotherapy alone may not be sufficient to increase discharge home. Its effectiveness depends also on the availability of appropriate discharge destinations, family willingness and capacity to support patients at home and timely access to community-based rehabilitation services.

Physiotherapist workforce capacity could be expanded, but the Italian system faces the additional paradox that enhanced in-hospital physiotherapy may not produce the primary benefit sought, i.e. earlier discharge home, if patient and family preferences continue to favour institutional transfer. The main outcome measure of the INITIATE trial is days at home within 30 days: it assumes that patients actually want to return home and can do so. This is not necessarily true in the Italian context: institutional transfer is a freely exercised right regardless of clinical status, and DAH30 may systematically underperform as an outcome measure. Encouraging and extending early rehabilitation could reduce the need for it later only if the destination after acute care is the patient’s usual residence, a condition that Italian policy, culture and patient expectation currently conspire against.

The WHiTE 15: INITIATE trial represents exactly the kind of rigorous, large-scale, pragmatic evidence generation needed in this field: it will inform future guideline revisions and shape rehabilitation practice in the UK and beyond. Yet its direct translatability to the Italian context is constrained by several structural problems: a chronic SSN physiotherapy workforce deficit that would make INITIATE-level intensification prohibitively expensive; a regulatory framework that systematically decouples discharge destination from clinical recommendation; and strong social and cultural preferences for institutional care rather than returning home after hip fracture surgery. Addressing these barriers will require a research agenda specifically designed for the Italian setting: studies investigating the psychosocial and family-level determinants of discharge preferences; cost-effectiveness analyses of early supported discharge programmes adapted to Italian community services; and policy interventions that reframe institutional transfer from a default entitlement to a clinically indicated exception.

Until these structural questions are confronted, the most we can hope for is that the lessons from INITIATE will prompt the various stakeholders to engage in an honest discussion that Italy urgently needs.

## Data Availability

Not applicable. No datasets were generated or analysed for this editorial.

## Declarations

### Use of AI

Language editing was performed using Grammarly (Superhuman Platform Inc., San Francisco, CA, USA).

## References

[1] Kearney RS, Costa ML (2026) Increasing mobility in hospital after hip fracture. Bone Joint J 108-B(6):747–750 10.1302/0301-620X.108B6.BJJ-2026-0017.R1 42219168

[2] Management of hip fracture in adults: summary of NICE guidance (2011) BMJ 342:d3304. 10.1136/bmj.d3304 21693526

[3] Fernandez MA, Arnel L, Gould J, McGibbon A, Grant R, Bell P, White S, Baxter M, Griffin X, Chesser T, Keenm D, Kearney RS, White C, Costa ML (2018) Research priorities in fragility fractures of the lower limb and pelvis: a UK priority setting partnership with the James Lind Alliance. BMJ Open 8(10):e023301 10.1136/bmjopen-2018-023301 PMC6194466 30287674

[4] Eurostat. Population structure and ageing. Statistics Explained. https://ec.europa.eu/eurostat/statistics-explained/index.php?title=Population_structure_and_ageing. Accessed Apr 2026.

[5] Silveri MC, Bruni G, D’Angelo D et al (2021) Trends in hip and distal femoral fracture rates in Italy from 2007 to 2017. Bone 145:115833 10.1016/j.bone.2020.115752 33188958

[6] AGENAS. Programma Nazionale Esiti 2025. Report su dati 2024. Roma: Agenzia Nazionale per i Servizi Sanitari Regionali; 2025

[7] Dettoni F, Peveraro A, Dettoni A, Rossi R, Castoldi F, Zareh A, Amberti F, Giai Via A, Bonasia D (2011) Epidemiology of hip fractures in northwestern Italy: a multicentric regional study on incidence of hip fractures and their outcome at 3-year follow-up. Musculoskelet Surg. 10.1007/s12306-011-0159-y 21769597

[8] Maffulli N, Aicale R (2022) Proximal femoral fractures in the elderly: a few things to know, and some to forget. Medicina (Kaunas) 58(10):1314 10.3390/medicina58101314 PMC9612001 36295475

[9] Kanis JA, Odén A, McCloskey EV, et al; on behalf of the IOF Working Group on Epidemiology and Quality of Life. A systematic review of hip fracture incidence and probability of fracture worldwide. Osteoporos Int 2012;23:2239–2256. 10.1007/s00198-012-1964-3 PMC3421108 22419370

[10] Chen YP, Kuo YJ, Hung SW et al (2021) Loss of skeletal muscle mass can be predicted by sarcopenia and reflects poor functional recovery at one year after surgery for geriatric hip fractures. Injury 52(11):3446–3452 10.1016/j.injury.2021.08.007 34404509

[11] Li Y, Wang T, Zhang J (2025) Intra-operative blood transfusions raise the risk of postoperative delirium and impede functional recovery in elderly hip fracture patients: a propensity score-matched study. J Orthop Traumatol 26:12 10.1186/s10195-025-00825-2 PMC11871276 40021592

[12] Mazarello Paes V, Ting A, Masters J et al (2025) A systematic review of evidence regarding the association between time to mobilization following hip fracture surgery and patient outcomes. Bone Jt Open 6(7):741–747 10.1302/2633-1462.67.BJO-2024-0243.R1 PMC12221528 40602785

[13] Crotty M, Whitehead CH, Gray S, Finucane PM (2002) Early discharge and home rehabilitation after hip fracture achieves functional improvements: a randomized controlled trial. Clin Rehabil 16(4):406–413 10.1191/0269215502cr518oa 12061475

[14] Holzapfel DE, Kappenschneider T, Holzapfel S et al (2025) Intensive care needs after hip and knee replacement: understanding risk profiles for severe postoperative complications. J Orthop Traumatol 26:42 10.1186/s10195-025-00862-x PMC12229386 40608156

[15] Zidén L, Frändin K, Kreuter M (2008) Home rehabilitation after hip fracture. A randomized controlled study on balance confidence, physical function and everyday activities. Clin Rehabil 22(12):1019–1033 10.1177/0269215508096183 19052241

[16] Zidén L, Kreuter M, Frändin K (2010) Long-term effects of home rehabilitation after hip fracture - 1-year follow-up of functioning, balance confidence, and health-related quality of life in elderly people. Disabil Rehabil 32(1):18–32 10.3109/09638280902980910 19925273

[17] Levi Y, Punchik B, Zikrin E et al (2020) Intensive inpatient vs. home-based rehabilitation after hip fracture in the elderly population. Front Med 7:592693 10.3389/fmed.2020.592693 PMC7581791 33163503

[18] Tedesco D, Gibertoni D, Rucci P et al (2018) Impact of rehabilitation on mortality and readmissions after surgery for hip fracture. BMC Health Serv Res 18(1):701 10.1186/s12913-018-3523-x PMC6131904 30200950

[19] Pitzul KB, Wodchis WP, Kreder HJ, Carter MW, Jaglal SB (2017) Discharge destination following hip fracture: comparative effectiveness and cost analyses. Arch Osteoporos 12(1):87 10.1007/s11657-017-0382-8 28965297

[20] Handoll HHG, Cameron ID, Mak JCS, Panagoda CE, Finnegan TP (2021) Multidisciplinary rehabilitation for older people with hip fractures. Cochrane Database Syst Rev 11:CD007125 10.1002/14651858.CD007125.pub3 PMC8586844 34766330

[21] Karlsson Å, Berggren M, Gustafson Y, Olofsson B, Lindelöf N, Stenvall M (2016) Effects of geriatric interdisciplinary home rehabilitation on walking ability and length of hospital stay after hip fracture: a randomized controlled trial. J Am Med Dir Assoc 17(5):464.e9-464.e15 10.1016/j.jamda.2016.02.001 26975205

[22] Berggren M, Karlsson Å, Lindelöf N et al (2019) Effects of geriatric interdisciplinary home rehabilitation on complications and readmissions after hip fracture: a randomized controlled trial. Clin Rehabil 33(1):64–73 10.1177/0269215518791003 PMC6311618 30064264

[23] Karlsson Å, Lindelöf N, Olofsson B et al (2020) Effects of geriatric interdisciplinary home rehabilitation on independence in activities of daily living in older people with hip fracture: a randomized controlled trial. Arch Phys Med Rehabil 101(4):571–578 10.1016/j.apmr.2019.12.007 31935353

[24] Lake D, Kumar S, Geng F, Gozalo P (2025) Comparative effectiveness of inpatient rehabilitation versus skilled nursing facilities for stroke and hip fracture patients. J Am Geriatr Soc 74(1):64–73 10.1111/jgs.70164 PMC13077050 41129694

[25] Aletto C, Aicale R, Pezzuti G, Bruno F, Maffulli N (2020) Impact of an orthogeriatrician on length of stay of elderly patient with hip fracture. Osteoporos Int 31(11):2161–2166 10.1007/s00198-020-05510-0 32572520

[26] Quaranta M, Miranda L, Oliva F, Migliorini F, Pezzuti G, Maffulli N (2021) Haemoglobin and transfusions in elderly patients with hip fractures: the effect of a dedicated orthogeriatrician. J Orthop Surg Res 16(1):387 10.1186/s13018-021-02524-0 PMC8207795 34134743

[27] Santacaterina F, Miccinilli S, Sterzi S et al (2023) Rehabilitation after hip fracture surgery: a survey on Italian physiotherapists’ knowledge and adherence to evidence-based practice. Healthcare (Basel) 11(6):799 10.3390/healthcare11060799 PMC10048225 36981456

[28] Abrahamsen C, Zindy M, Damsgaard EM et al (2022) Patient perspectives of recovery after hip fracture: a systematic review and qualitative synthesis. Disabil Rehabil 44(22):6631–6641 10.1080/09638288.2021.1965228 34428389

[29] Müller, F., Zellner, M., Bäuml, C. Risk factors for surgical site infection following treatment of proximal femoral fracture: a matched-pair analysis (2025). J Orthop Traumatol 26, 35 10.1186/s10195-025-00856-9 PMC12137835 40464828

[30] Magaziner J, Simonsick EM, Kashner TM et al (1989) Determinants of recovery 12 months after hip fracture: the importance of psychosocial factors. Am J Public Health 79(3):279–286 10.2105/ajph.79.3.279 PMC1349547 2916712

[31] The PIPPAS Study Group (2024) Optimizing periprosthetic fracture management and in-hospital outcome: insights from the PIPPAS multicentric study of 1387 cases in Spain. J Orthop Traumatol 25:13 10.1186/s10195-024-00746-6 PMC10920552 38451303

[32] Bacci C, Longobardi G, Viora L et al (2024) Association between patient activation level and functional outcomes in older adults with hip fractures. Arch Gerontol Geriatr 124:105472 10.1016/j.archger.2024.105472 38728823

[33] Key-Stone Research & Consulting. Figures of physiotherapy in Italy. https://www.key-stone.it/figures-of-physiotherapy-in-italy/. Accessed Apr 2026.

[34] Eurostat. Healthcare personnel statistics – dentists, pharmacists and physiotherapists. Statistics Explained. 2025. https://ec.europa.eu/eurostat/statistics-explained/index.php?title=Healthcare_personnel_statistics_-_dentists,_pharmacists_and_physiotherapists. Accessed Apr 2026.

